# Supplementary material for: Impact of intensive adherence counseling on viral load suppression and mortality among people living with HIV in Kampala, Uganda: A regression discontinuity design
Source: PLOS Glob Public Health. 2023 Aug 7;3(8):e0002240. doi: 10.1371/journal.pgph.0002240 (PMC10406184; doi:10.1371/journal.pgph.0002240)
Supplement: Attachment — Submitted filename: Response to Reviewers.docx [file pgph.0002240.s004.docx]

**Point by point response**

**Journal Requirements:**

1. Please include a complete copy of PLOS’ questionnaire on inclusivity in global research in your revised manuscript. Our policy for research in this area aims to improve transparency in the reporting of research performed outside of researchers’ own country or community. The policy applies to researchers who have travelled to a different country to conduct research, research with Indigenous populations or their lands, and research on cultural artefacts. The questionnaire can also be requested at the journal’s discretion for any other submissions, even if these conditions are not met. Please find more information on the policy and a link to download a blank copy of the questionnaire here: https://journals.plos.org/globalpublichealth/s/best-practices-in-research-reporting. Please upload a completed version of your questionnaire as Supporting Information when you resubmit your manuscript.

**Response: Thank you for this comment. We have downloaded and filled the PLOS’ questionnaire on inclusivity in global research. The complete version of the questionnaire has been uploaded as a supporting information, marked S2 File. In the manuscript, we have included the questionnaire under the supporting information marked as “S2 File. Checklist”.**

**As required, in the methods section, we have included the questionnaire under the heading “Inclusivity in global research”. The new sentence under this heading reads: “Additional information regarding the ethical, cultural, and scientific considerations specific to inclusivity in global research is included in the Supporting Information (S2 File).”**

2. Please review your reference list to ensure that it is complete and correct. If you have cited papers that have been retracted, please include the rationale for doing so in the manuscript text, or remove these references and replace them with relevant current references. Any changes to the reference list should be mentioned in the rebuttal letter that accompanies your revised manuscript. If you need to cite a retracted article, indicate the article’s retracted status in the References list and also include a citation and full reference for the retraction notice.

**Response: Thank you. We have reviewed the references and do confirm that they are complete and correct, and that no retracted articles have been cited.**

3. Additional Editor Comments (if provided):

**Response: No additional editor comments provided. Thank you.**

4. Reviewers' comments

**Response: No additional reviewer comments have been provided. We thank the reviewers for the efforts.**
