# Supplementary material for: Impact of intensive adherence counseling on viral load suppression and mortality among people living with HIV in Kampala, Uganda: A regression discontinuity design
Source: PLOS Glob Public Health. 2023 Aug 7;3(8):e0002240. doi: 10.1371/journal.pgph.0002240 (PMC10406184; doi:10.1371/journal.pgph.0002240)
Supplement: Attachment — Submitted filename: Responses to Reviewer comments.docx [file pgph.0002240.s003.docx]

**Peer Review Comments**

Dear reviewers,

We thank you for reviewing our manuscript and for providing us with wonderful comments which have helped to improve the quality of the manuscript. We have responded to each of the comments and we look forward to receiving a favorable response.

# Reviewer #1

Overall, this is a well-written paper, therefore, my review outcomes are suggestions some of which may not be adopted should the authors see it fit.

1. Abstract: Overall, the abstract provides a clear and concise summary of the study. Here are some specific comments for improvement:

**Response: Thank you for the wonderful comments. We wish to address all the comments regarding the abstract but we have word limitations. The journal guideline states that “The Abstract should be succinct; it must not exceed 300 words. Authors should mention the techniques used without going into methodological detail and should summarize the most important results.” In certain sections of the abstract, we have addressed the comments amidst the tight word count.**

- The abstract does not provide enough context for the problem being studied. It would be helpful to provide some background information on the issue of HIV and ART adherence, as well as the prevalence of VL ≥1,000 copies/ml in the study population.

**Response: Thank you. We have not included this information due to word count but it has been addressed in the main body of the manuscript. We propose to maintain the current description.**

- The abstract could benefit from a clearer description of the study design and methods. Specifically, it would be helpful to explain the regression discontinuity design in layman's terms and why it was appropriate for this study.

**Response: We agree to describing the design in a simplified manner. We have revised the design description to read as follows: “We utilized a regression discontinuity design, a quasi-experimental method for effect estimation when interventions depend on a cut-off. PLHIV just above VL ≥1,000 copies/ml cut-off who received ≥3 IAC sessions formed the intervention group while those just below the cut-off who received routine psychosocial support constituted the control group”.**

- The results are clearly presented, but it would be helpful to provide some explanation for why the odds of repeat VL suppression were lower in the intervention group. Were there any potential reasons for this finding that were discussed in the study? Additionally, it would be helpful to mention the effect size of the intervention (i.e., the actual difference in VL suppression rates between the control and intervention groups).

**Response: Thank you. We did mention the proportions of VL suppression within the optimal bandwidth between the intervention and control arm, which was 85.7% versus 98.6% respectively. We have stated the magnitude of the intervention effect on VL suppression which reads: "In multivariable analysis, the odds of repeat VL suppression were 91% lower in the intervention than in the control group (OR 0.09; 95% CI, 0.01ꟷ0.66)."** **We found it impractical to provide some explanations as to why the odds of repeat VL suppression were lower in the intervention group in the abstract due to word count restrictions but we did so in the main discussion section of the manuscript.**

- The abstract ends with some useful recommendations for future research, but it would be helpful to provide some more context for why investigating the fidelity of IAC implementation and reasons for VL persistence are important.

**Response: Implementation fidelity is critical in successfully translating evidence-based interventions into practice. We recognize that the context within which IAC is being implemented influences VL suppression and understanding it is important. Our view is that there could be inadequacies that warrants further research. We have added a sentence to explain why it is important to investigate the fidelity of IAC implementation. The revised sentence reads: "Findings suggest a need to investigate the IAC implementation fidelity for successful translation in practice and the reasons for VL persistence beyond the suppression threshold."**

2. Background: The background provides a clear overview of the issue of viral load suppression in people living with HIV (PLHIV) and the use of intensive adherence counseling (IAC) to improve adherence to ART. It summarizes existing evidence on the effectiveness of IAC, highlighting mixed results from previous studies. The authors note the lack of causal evidence on the effect of IAC on viral load suppression, which the EFFINAC study aims to address. Overall, the background provides relevant and concise information to contextualize the study and the research question. However, some additional information on the importance of viral load suppression and the impact of unsuppressed viral loads on PLHIV's health outcomes could further strengthen the background.

**Response: Thank you for the comment. We have added a new sentence in the background regarding the importance of VL suppression and its impact on health outcomes and it reads: “VL suppression reduces vulnerability to opportunistic infections, improves the quality and quantity of life of PLHIV, reduces the risk of HIV transmission during sexual intercourse, prevents mother to child transmission of HIV during labor, delivery, and breastfeeding, and it is indicative of an optimally performing HIV control program.”**

3. Study design: The study design presented, regression discontinuity design (RDD), appears appropriate for evaluating the causal effect of IAC on VL suppression and all-cause mortality. The use of RDD is justified since intervention assignment depends on a cut-off, with IAC being provided to PLHIV with unsuppressed VL (VL ≥1,000 copies/ml), while those with suppressed VL receive routine psychosocial support (PSS). RDD is suitable when there is a discontinuity in the probability of treatment assignment at a certain threshold, and participants just above and below this threshold are comparable. The study appears to meet the RDD conditions, with VL serving as the assignment variable, the cut-off being VL ≥1,000 copies/ml, and outcomes being measured for all participants regardless of intervention.

**Response: Thank you for the positive feedback.**

4. Study population and other sections through measurements: Overall, the description of the study population and measurements is comprehensive and clear

**Response: Thank you for this compliment.**

5. Study limitation: Could this be a potential limitation of the study design; that it assumes that individuals just above and below the cut-off are similar in both measured and unmeasured confounders, which may not always be the case. Additionally, the study design cannot account for potential selection bias, where individuals with different characteristics are more likely to receive IAC based on other factors not accounted for in the RDD. Therefore, it is important to interpret the findings of the study with caution and consider these potential limitations in the interpretation of the results.

**Response: The use of RDD significantly reduces selection bias by restricting the data analysis to a small proportion of the sample, just above and just below the cut-off. Within this bandwidth, both measured and unmeasured covariates are balanced and we demonstrated this covariate balance in the study in Table 2. By achieving covariate, RDD removes selection bias and emulates a randomized controlled trial or RCT. We elaborately described the advantages and applications of the RDD under study design and its limitations in the discussion section earlier. We agree that the study demonstrated covariate balance for only measured covariates although the potential for balance in unmeasured covariates is equally likely. We did acknowledge the limitations regarding unmeasured covariates earlier and the sentence read: “Limitations of the study included unmeasured confounders due to the use of observational data.”**

6. Discussion: Overall, this discussion provides a well-organized and informative critique of the effectiveness of IAC in achieving VL suppression among PLHIV in Uganda and similar settings in sub-Saharan Africa. The discussion cites relevant studies and provides plausible explanations for the lower likelihood of repeat VL suppression despite IAC. Additionally, the implications of the findings for practice, policy, and future research are well-articulated. However, there are a few areas where the discussion could be improved. Firstly, the discussion could benefit from a more detailed explanation of what IAC is and how it works to support ART adherence. This would help readers who are not familiar with the intervention to better understand the findings and implications of the study. Secondly, the discussion could have been more specific in identifying the limitations of the study, such as the small sample size, the lack of randomization, and the potential for confounding variables. By acknowledging these limitations, the authors could have enhanced the credibility and transparency of their study. Finally, the discussion could have provided more concrete recommendations for how IAC implementation could be improved. While the authors suggest that the fidelity, dose, and intensity of IAC implementation need rigorous evaluation in future research, they do not provide specific suggestions for how these improvements could be made. Providing more specific recommendations could help guide policy and practice in a more tangible way. Overall, while the discussion provides a thorough and insightful critique of the study's findings, there are opportunities for improvement in terms of providing more detailed explanations, acknowledging limitations, and offering specific recommendations for future research and practice.

**Response: We thank you for the wonderful comments. We agree to provide a detailed description of IAC in the discussion. However, we had already provided this description in the methods section under the heading “measurements”. We are of the view that further description in the discussion section would be a repeat. We propose to main the description only in the methods section.**

**We have added a sentence to indicate that the small sample size within the optimal bandwidth is a limitation and it reads: "Our sample size within the optimal bandwidth is relatively small so studies with a large sample size would be needed to provide additional evidence".**

**We agree that confounding and the lack of randomization are key limitations in observational studies but this has been mitigated by the use of an RDD. The issue of fidelity, intensity, dose-response, and concrete recommendations as to how IAC could be improved are being tackled in a follow-up paper that we are working on at the moment.**

# Reviewer #2:

1. I did not find any statement about availability of data. Otherwise the manuscript highlights an important concern in HIV treatment and care. It is well written and all analyses are well done. What is not clear is how the participants in the control group that had unsuppressed viral load at the end of the study were handled. The inclusion criteria is also silent about the duration on ART for the participants included in the study. My major concern is how receiving IAC was measured to conclude that all participants in the intervention arm received at least 3 sessions of IAC, yet this being a retrospective data analysis using routinely collected data. I would expect some level of missingness both in IAC and viral load results.

**Response: Thank you for the comment. The data analyzed have been made available as supporting information (S1 File). And, If a participant in the control group does not achieve VL suppression (unsuppressed VL) after psychosocial support, the HIV treatment guideline recommends IAC. We have included this information and it reads: “In the event of unsuppressed VL after PSS, IAC is provided as recommended.”**

**The duration of ART for inclusion in either the intervention or control group was six months and this was earlier highlighted under the description of the intervention. The sentence reads: "IAC refers to a psychosocial counselling and support intervention provided by a multidisciplinary team of health workers (clinicians, nurses, counsellors, family members, and peers among others) to PLHIV with unsuppressed VL after six or more months of ART".**

**Regarding the measurement of IAC, all PLHIV receiving IAC are registered in an MoH-provided IAC register to ease the tracking of IAC sessions. We had initially described this in the study population section as “Additional data for PLHIV receiving IAC were abstracted from the IAC register”. We have now provided the description again under the intervention section and it reads: “All IAC sessions received are reported in the MoH IAC register to ease the tracking of the sessions per individual.**

**And, yes, there was some level of missingness that we reported earlier in the analysis section and the sentence reads: “Two variables, MUAC and baseline weight, had missing observations which were imputed using the multivariate imputations via the chained equations (MICE)”.**

**Overall, the study sites have a robust HIV information management system and benefit from periodic data audits and technical support and assistance in data management from the Infectious Diseases Institute (IDI) that we have already described under the study setting section. So, the data quality is generally good.**
